# Direct Determination of Ratios of All Conformations and Their Lifetimes for Small Flexible Molecules from Molecular Dynamics Simulations: 1,3-Propanediol in an Aqueous Environment

**DOI:** 10.3390/molecules30061285

**Published:** 2025-03-13

**Authors:** Olga V. Grineva

**Affiliations:** Chemistry Department, Moscow M. V. Lomonosov State University, 119991 Moscow, Russia; ovg@phys.chem.msu.ru

**Keywords:** aliphatic diol, aqueous solution, conformational equilibrium, lifetimes of conformations, hydrogen bond

## Abstract

For the first time in the course of molecular dynamics modeling of a liquid, the conformations of each of the small flexible molecules present in the system were fixed at short (1 ps) time intervals. This allowed the establishment of the ratios between various individual conformations and their families and determination of the average lifetimes of both individual conformations and families. As an example, data are presented for modeling boxes with different numbers of molecules (800, 2700, and 6400) for an aqueous solution with 1 mol. % 1,3-propanediol at 298.15 K and 1 atm. The results of the conformational analysis turned out to be very close for systems with different numbers of molecules and with different choices of initial conformations. For the systems under investigation, the tTTg conformation, which does not have intramolecular hydrogen bond, predominated (37–39%), and the total fractions of all conformations in the TT family were 74–76%. Only 0.4–0.5% of 1,3-propanediol molecules had conformations with the possible formation of intramolecular hydrogen bond, although the most stable conformers of free 1,3-propanediol molecules exhibit such a bond. The average lifetimes of each individual conformation did not exceed 7 ps in simulated systems, while the maximum lifetimes reached 60 ps. The average lifetimes of the main chain vary from ~110 ps in TT family to ~12 ps in GG′ family, in which the conformations tend to have intramolecular hydrogen bonds. It was found that calculations for an individual 1,3-propanediol molecule at the MP2/aug-cc-pVDZ or MP2/aug-cc-pVTZ theoretical levels lead to 22 conformers both in vacuum and by using the PCM model for implicit aqueous solvation (at the MP2/aug-cc-pVDZ level) and that such solvation reduces the energy difference between the conformers.

## 1. Introduction

Most organic molecules are not rigid and can assume several conformations. Since conformational changes in protein molecules are an integral part of many biochemical processes, including their interactions with drugs [1], a considerable amount of work has been conducted in this field, in particular using the molecular dynamics method [2,3,4].

However, an equally important task is studying the conformations of small flexible molecules depending on factors related both to the structure of the molecules themselves and to external conditions, in order to develop the theoretical foundations of conformational analysis. Realization of conformations of free molecules depends on intramolecular interactions only, while in condensed phases, both intra- and intermolecular interactions influence the conformational composition. The hydrogen bond (H-bond for short), as the strongest type of non-covalent interaction, tends to have the strongest influence. Therefore, of particular interest are molecules for which, depending on the environment, the conformations can be stabilized either by intra- or intermolecular hydrogen bonds and, possibly in some cases, a combination thereof.

Experimental fixation of minor conformations often turns out to be impossible even when studying free molecules, and even more so in liquids; therefore, modeling plays an important role in the study of molecular conformations in liquids. As a result of studying [5] the 1,3-butanediol–water–acetonitrile system using several methods, including molecular dynamics modeling, it was shown that the preferred conformations of the 1,3-butanediol molecule change depending on the ratio of water and acetonitrile concentrations. If the concentration of water is large, conformations that favor the formation of intermolecular hydrogen bonds predominate, and if the concentration of acetonitrile is large, then conformations with intramolecular hydrogen bonds predominate. However, in this useful work the authors did not take advantage of one of molecular dynamics modeling primary strengths, namely, the ability to accurately determine the conformations of molecules from the coordinates of atoms. They limited themselves to considering the radial distribution functions for several pairs of atoms, which is a typical approach in such studies. There is definitely a correlation between the average intramolecular interatomic distances and the conformations of the molecule. However, this is not ideal (see Section 3.2), and, most importantly, the radial distribution functions do not reflect time-related parameters (the lifetimes of individual conformations, etc.) in principle.

Thus, the aim of this work was to try out another method for determining conformations of small molecules—namely, fixing the coordinates of all target molecules in the system under consideration at short time intervals. The subsequent analysis should have made it possible to calculate the fraction of all conformations of these molecules and their lifetimes. 1,3-propanediol was chosen as the target molecule, and a dilute aqueous solution was chosen as the system under consideration. 1,3-propanediol and its aqueous solutions are used widely [6,7] and are also of interest in biomimetics. The mutual arrangement of hydroxyl groups in this compound is the same as in 1,3-butanediol, so it can participate in both the formation of intra- and intermolecular hydrogen bonds, but the 1,3-propanediol molecule has significantly fewer geometrically different conformations (due to the smaller number of carbon atoms and the symmetrical equivalence of some of the conformations), which was more convenient for a complete conformational analysis.

In addition to the main task associated with the development of a method for studying the conformations of flexible molecules based on molecular dynamics modeling data, it was also of interest to compare the characteristic set of conformations of 1,3-propanediol in dilute aqueous solutions of this compound with its conformation in crystalline phase [8] and preferred conformations for an individual molecule [9,10,11,12,13,14,15,16,17,18,19].

## 2. Results

### 2.1. Quantum Chemical Calculations

In recent decades, quantum chemical calculations have been the usual starting point for conformational analysis of compounds in various systems. The aim of quantum chemical calculations in this work was to specify the number of 1,3-propanediol conformers in vacuum, as well as to clarify the effect of implicit aqueous solvation on the results.

**Figure 1:**
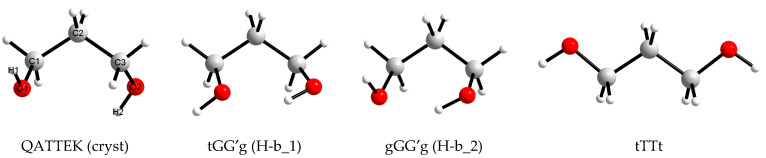
Views and designations of 1,3-propanediol conformations used in this work to create initial boxes for molecular dynamics modeling.

The possibility of the existence of the 1,3-propanediol molecule in different conformations is due to the fact that the torsion angles H1−O1−C1−C2, O1−C1−C2−C3, C1−C2−C3−O2, and C2−C3−O2−H2 (Figure 1) can have different values.

It is known that for conformers corresponding to the energy minima for short linear aliphatic molecules (e.g., *n*-butane), the values of analogous angles are close to 60° (G), 180° (T) or −60° (G′). A sequential variation of three values for four angles yields 81 combinations, but for 1,3-propanediol molecules, most of them lead to identically or mirror-equivalent conformations, and only 25 variants are unique. Traditionally, when designating conformations of aliphatic diols, torsion angles that include hydroxyl H atoms are denoted by lowercase letters, as in Figure 1.

Quantum chemical studies of 1,3-propanediol were started in the late 1980s [14]. In two studies [14,16], optimizations of all 25 conformations created in the manner described above were performed. According to current views, the theoretical levels of calculations in both studies were very low (HF/4-21G [14] and HF/4-31+G(d) [16]); however, the authors of [16] obtained an important result: not all 25 initial conformations of 1,3-propanediol lead to conformers (i.e., conformations corresponding to energy minima). In Ref. [16], 23 conformers were found. In Ref. [15], performed at about the same time, the authors supplemented the initial conformations by specifying *cis*-arrangements of H atoms in hydroxyl groups. They used several semiempirical and molecular mechanical methods for calculations, but did not report the number of conformers obtained by each method. In a number of later works [11,12,17,18], quantum chemical calculations were performed only for some of the possible initial conformations. In Ref. [17], where the theoretical level B3LYP/6-311++G(2d,2p) was used, the authors noted the absence of one conformer, which according to [16] was stable, but in the text, they gave an erroneous designation for it. For the conformation of gGG′g indicated by them, a stable conformer undoubtedly exists, and it was presented by the authors in the Table 1 [17]. In Ref. [19], all 25 initial conformations were optimized in two ways (B3LYP/6-31+G(d,p) and MP2/6-311++G(d,p)), and as a result, the authors obtained 23 conformers in agreement with Ref. [16]. In all the studies mentioned in this section, the calculations were performed for 1,3-propandiol molecules in a vacuum.

In this work, calculations in vacuum were made with the MP2 [20,21] method using two basis sets (aug-cc-pVDZ [22,23] and aug-cc-pVTZ [22,23]). From the data presented in Table 1, it follows that the basis set expansion did not have a noticeable effect on the results, but calculations (especially of frequencies) with the aug-cc-pVTZ basis set take significantly more time. Therefore, calculations within PCM model [24] for implicit aqueous solvation were performed only with the aug-cc-pVDZ basis set.

**Figure 2:**
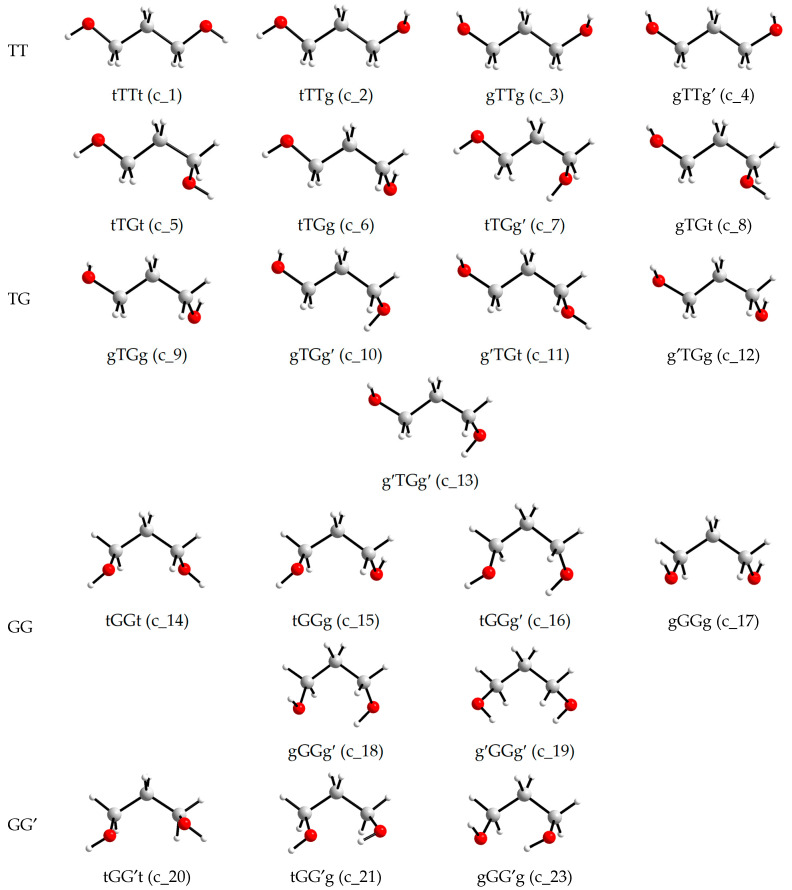
Conformers of an individual 1,3-propanediol molecule (i.e., conformations corresponding to energy minima) according to the MP2/aug-cc-pVDZ or MP2/aug-cc-pVTZ optimizations.

**Figure 3:**
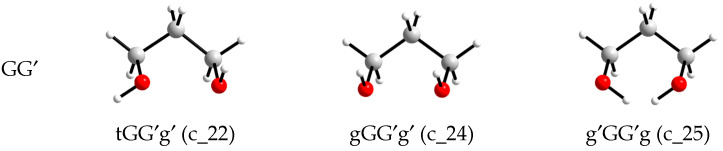
Conformations of an individual 1,3-propanediol molecule that transform into conformers with other designations upon the MP2/aug-cc-pVDZ or MP2/aug-cc-pVTZ optimizations.

The views of the conformers are shown in Figure 2, and the views of the conformations that transform into conformers with other designations during optimization are shown in Figure 3. It is seen that the transitions to conformers with other designations are associated with a substantial change in one of the torsion angles involving a hydroxyl hydrogen atom, to form an intramolecular hydrogen bond. In the initial conformations, either both hydroxyl hydrogens are not turned to the oxygens of the neighboring hydroxyl groups (c_22 and c_24) or, conversely, both are turned (c_25), which also does not facilitate the formation of a hydrogen bond.

### 2.2. Comparison of Density Values Calculated from Molecular Dynamics Simulations with Experimental Ones

As stated in the Section 1, the main objective of this work was to perform conformational analysis of 1,3-propanediol molecules in a dilute aqueous solution using directly the atomic coordinates obtained during molecular dynamics simulations. Since this publication presents such an approach for the first time, it was decided to limit the consideration to one point of the phase diagram (i.e., not to vary the solution concentration, temperature, and pressure), but to analyze the influence of the choice of the initial conformation, the size of the model system, and the trajectory length (with the same time step for different model systems), and the total number of conformations considered for each system on the final results.

To set the initial molecular structure of target compound when creating boxes for modeling liquids, they most often use (a) data on the molecular structure of this compound in crystals (if any); (b) data on the structure of free molecules obtained experimentally or with quantum chemical calculations; (c) the simplest form of the molecule (it is often offered by visualizers of structural formulas). In this work, all three of the listed options were used (Figure 1).

The Cambridge Crystallographic Database [25] contains data on homomolecular crystals of 1,3-propanediol (reference code QATTEK). The molecular conformation in this substance can be denoted as gGGg′, but its geometric parameters differ markedly from those obtained as a result of optimization by the MP2/aug-cc-pVDZ or MP2/aug-cc-pVTZ methods in this work (Table 2). During optimization, oxygen atoms, initially located farther away, approach each other, which leads to the formation of an intramolecular H-bond, although it turns out to be longer (~3.1 Å) than the classical one (~2.8 Å, as in conformers c_21 and c_23). Each molecule in QATTEK takes part in the formation of four intermolecular hydrogen bonds, and there is no intramolecular hydrogen bond [8,26].

On the contrary, for a free 1,3-propanediol molecule, experimental [9,10,11,12,13] and computed [11,12,14,15,16,17,18,19] data indicated that the conformers with an intramolecular hydrogen bond were the most favorable, and the same result was obtained in this work (Table 1).

Visually, the simplest conformation of 1,3-propanediol is the fully elongated tTTt conformation (Figure 1), which does not have an intramolecular hydrogen bond, and this bond cannot be formed in it as a result of small changes in torsional angles.

Therefore, four variants of conformations were used in this work to create initial approximations (Table 3). More details on the formation of these systems are given in Section 4, Materials and Methods. Information about the program and the force fields used for simulations is also provided there.

To check the adequacy of these force fields, a comparison was made between the calculated and experimental density values (Table 4). The length of the trajectory can affect the values of the parameters being determined, and the number of points along it affects the accuracy, so the relevant information is also provided in the table.

The results for various systems used in MD simulations are in excellent agreement with each other, and their difference from the experimental value obtained in this work is 0.15–0.17%. This is a very good coincidence. It is an order of magnitude better than in the simulation of the water–1,3-butanediol system out of 1698 molecules (of which 6 molecules are 1,3-butanediol) [5], where the difference between the experimental and calculated density values was 1.6%.

### 2.3. Determination of Conformational Compositions of the Simulated Systems

In-house programs were compiled to determine the conformations of 1,3-propanediol from the data on the coordinates of atoms, which were recorded in the course of simulations with a step of 1 ps. For each torsion angle required to determine the conformation, the range of possible values (from 0 to 360°) was divided into three equal segments: the interval from 0 to 120° corresponded to the designation G/g, from 120 to 240°—T/t and from 240 to 360°—G′/g′.

**Figure 4:**
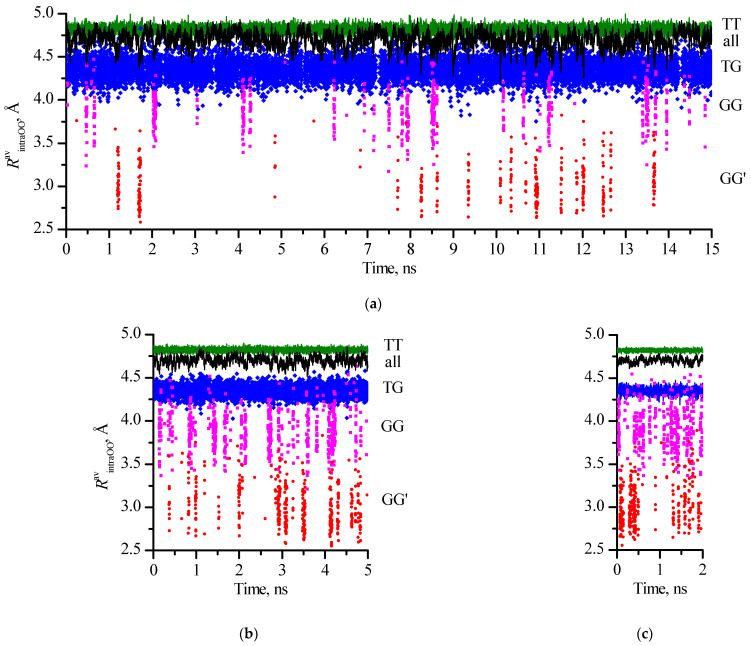
Distributions of O…O intramolecular distances in 1,3-propanediol molecules along molecular dynamics trajectories (averaged at each time point over the number of molecules in the particular families or the whole systems) for different size systems: 800_tTTt (**a**), 2700_cryst (**b**), and 6400_cryst (**c**).

Graphs showing the change in the conformational composition depending on the simulation time are presented for the systems with the different number of molecules in Figure 4.

To create these graphs, the conformation of each 1,3-propanediol molecule presented in the system was first assigned to one of four families (conformations that have the same designations for the torsion angles of the main chain, i.e., without taking into account the position of H in the hydroxyl groups). Then, for the conformations of each family, the intramolecular O…O distances were averaged for each time point recorded in the simulation. It was found that the values of *R*_intraOO_ formed rather wide corridors despite being averaged over the number of molecules. These intervals narrow as expected with an increase in the number of molecules in the system. Secondly, even in a large system (6400 molecules), the *R*^av^_intraOO_ values for the GG family at certain time intervals overlap with the *R*^av^_intraOO_ values for the TG and GG′ families. This clearly indicates that the instantaneous value of the distance O…O (as well as the value of any other intramolecular distance) is not an unambiguous indication of the conformation of molecules such as aliphatic diols in the liquid phase.

As seen from Table 5, the most common conformation in dilute aqueous solutions is the tTTg (c_2): its fraction is 37–39%. It should be noted that this fraction includes four symmetry-related variants of this conformation. Therefore, if we assume that there is a correlation between the prevalence and profitability of conformations, then the tTTt (c_1) conformation, which does not have symmetrically equivalent implementation options, is individually the most favorable. Most of the conformations belong to two families in which the formation of intramolecular hydrogen bonds is impossible: the fraction of conformations of the TT family was 74–76%, and the fraction of conformations of the TG family was 23–25%. Conformations with closely arranged hydroxyl groups (GG and GG′ families) were present in insignificant amounts (0.6–1.1% total for both families), while the fraction of conformations in which the mutual orientation of hydroxyl groups favors the formation of an intramolecular H-bond (c_16, c_18, c_21, c_23) was approximately two times less (0.4–0.5%)

### 2.4. Radial Distribution Functions

**Figure 5:**
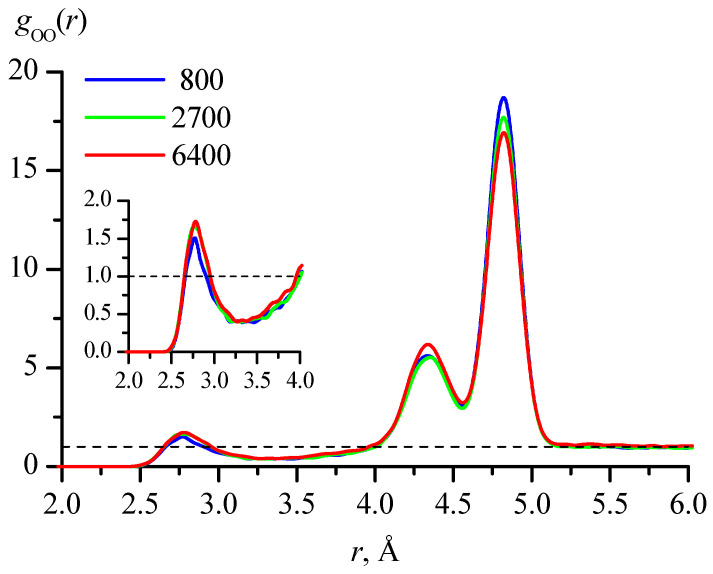
Radial distribution functions *g*_OO_(*r*), where O are oxygen atoms in hydroxyl groups, calculated for the 800_tTTt, 2700_cryst, and 6400_cryst systems with averaging of values obtained with 1 ps time step for trajectories of 10, 4, and 1 ns, respectively. The inset shows a larger view of the region corresponding to the first peak.

To compare the results of calculating the fractions of different conformations by the method proposed in this work with the traditionally used evaluation based on radial distribution functions, such functions were calculated. For the compound under consideration, the most characteristic for assessing conformations in dilute aqueous solutions is the distance between oxygens in two hydroxyl groups. Figure 5 shows the functions *g*_OO_(*r*) calculated for the 800_tTTt, 2700_cryst, and 6400_cryst systems with averaging the values obtained with 1 ps time step for trajectories of 10, 4, and 1 ns, respectively.

### 2.5. Lifetimes of Individual Conformations and Families of Conformations

Since the coordinates of the atoms were recorded with an interval of 1 ps, the minimum determined lifetime of the conformation is equal to this value. If the same conformation designation was retained for a given molecule at the next time step, its lifetime increased by 1 ps, and so on.

For conformations with contents of 1% or more (TT and TG families), the values of these parameters are perfectly reproduced in systems of different sizes and with different initial conformations of 1,3-propanediol molecules (Table 5). For conformations present in systems in very small amounts, the fluctuations of the parameters are naturally larger, but the results obtained for different systems are qualitatively similar.

**Figure 6:**
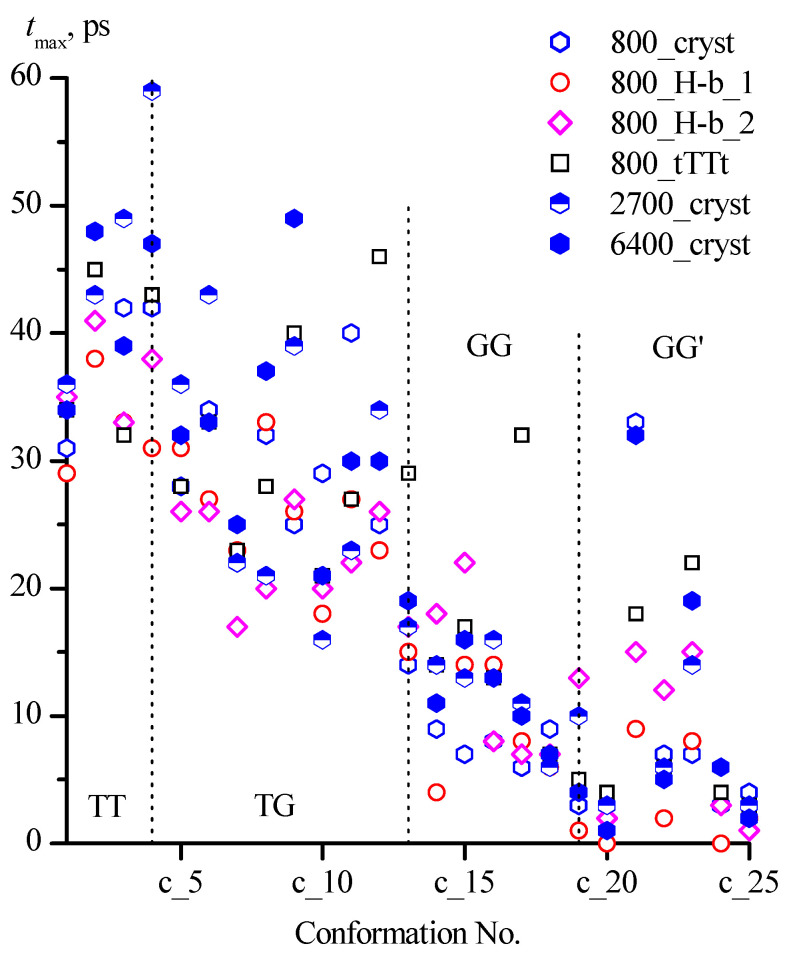
Maximum lifetimes of 1,3-propanediol conformations with different designations in systems under consideration.

The average lifetimes of all conformations did not exceed 7 ps, while the maximum lifetime reached 60 ps (Figure 6, Table 6).

Table 7 shows the average lifetimes of the configuration of the main chain of the 1,3-propanediol molecule in the considered aqueous solution (lifetimes for families of conformations).

## 3. Discussion

### 3.1. Quantum Chemical Calculations

In the present work, it was found that calculations at the theoretical levels MP2/aug-cc-pVDZ or MP2/aug-cc-pVTZ for the 1,3-propanediol molecule in vacuum lead to 22 conformers. Perhaps, a similar result could have been obtained earlier [17] using the B3LYP/6-311++G(2d,2p) calculation level, but the authors did not explicitly indicate the total number of conformers since they considered only the 10 most favorable ones and gave an erroneous designation for the missing conformer in the GG′ family.

For small alkanediol molecules, a reduction in the number of stable conformers compared to the number that would be obtained by sequential variation of *gauche*-*trans* values for torsion angles, and taking symmetry into account is typical [28,29]. As noted in Section 2.1, this is due to a change in the position of one of the hydroxyl hydrogen atoms to create an intramolecular hydrogen bond. From the available data, it follows that such a change in orientation is possible if the O and H atoms, potentially suitable for forming an H-bond, are not too far apart in the initial conformations (c_22, c_24, c_25, Figure 3). If the distance between them is large (c_20, Figure 2), the transformation does not occur, although the energy of the optimized conformer is the highest of all (Table 1).

**Figure 7:**
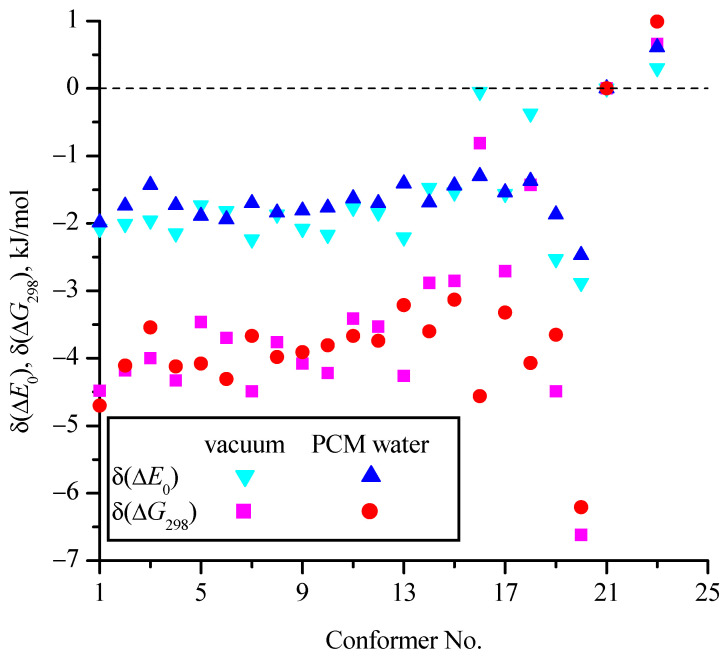
Change in relative (compared to c_21) energies of conformers with the zero-point corrections (δ(Δ*E*_0_) = Δ*E*_0_ − Δ*E*_el_) and thermal corrections to Gibbs energy (δ(Δ*G*_298_) = Δ*G*_298_ − Δ*E*_el_) for calculations performed at the MP2/aug-cc-pVDZ level in vacuum and using the PCM model for an implicit aqueous environment.

**Figure 8:**
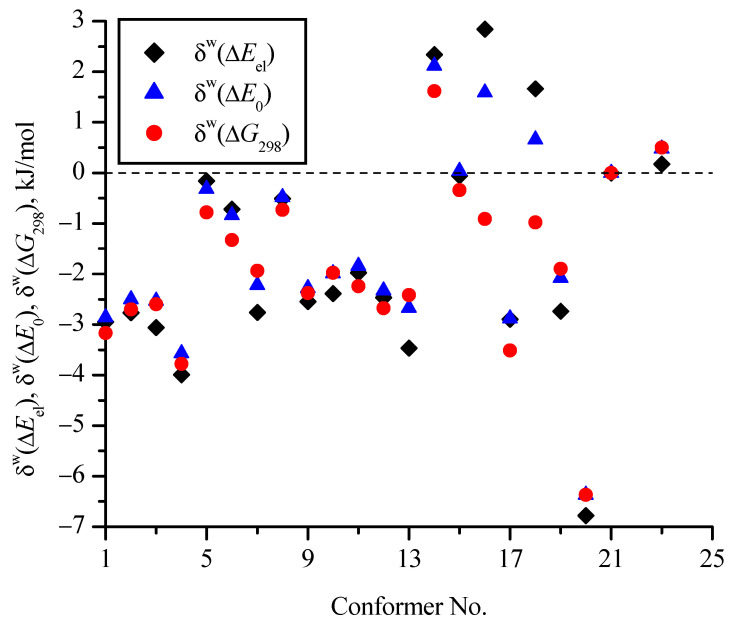
Change in relative (compared to c_21) energies of conformers when using the PCM model for an implicit aqueous environment compared to calculations in vacuum (δ^w^(Δ*E*_el_) = Δ*E*_el_^w^ − Δ*E*_el_^v^ and similarly for δ^w^(Δ*E*_0_) and δ^w^(Δ*G*_298_)) (all calculations at the MP2/aug-cc-pVDZ level).

Figure 7 and Figure 8 show the effect of the zero-point corrections and thermal corrections to Gibbs energy, as well as the implicit inclusion of the aqueous environment, on the relative (compared to c_21) energies of the conformers (Table 1). In Figure 7, the δ values correspond to changes in the relative energies of the conformers compared to Δ*E*_el_ (i.e., for a particular conformer δ(Δ*E*_0_) = Δ*E*_0_ − Δ*E*_el_ and δ(Δ*G*_298_) = Δ*G*_298_ − Δ*E*_el_). They are determined on the basis of MP2/aug-cc-pVDZ calculations in vacuum and within the PCM model for water. In Figure 8 values of δ^w^ show the change in the relative energies of the conformers (Δ*E*_el_, Δ*E*_0_, and Δ*G*_298_) when comparing calculations in a vacuum with calculations in PCM water on the MP2/aug-cc-pVDZ theoretical level (δ^w^(Δ*E*_el_) = Δ*E*_el_^w^ − Δ*E*_el_^v^ and similarly for δ^W^(Δ*E*_0_) and δ^W^(Δ*G*_298_)). It is seen that the zero-point corrections reduce the energy differences of most conformers relative to the conformer with the minimum energy (c_21) by 1.5−2 kJ/mol compared to Δ*E*_el_ for both calculations in vacuum and in aqueous medium within the PCM model. The thermal corrections to Gibbs energy reduce the differences compared to Δ*E*_el_ by 3–5 kJ/mol for most conformers. For the c_20 conformer, taking into account the corrections has the strongest effect on its relative energy (for example, δ(Δ*G*_298_) = −6.6 kJ/mol in vacuum), while for the c_16 and c_18 conformers in vacuum, these corrections have little effect. It is interesting that for the c_23 conformer, which is the second most favorable among all conformers, the corrections slightly (up to 1 kJ/mol) increase the difference in its energy from the energy of the most favorable conformer c_21.

The effect of PCM water on the relative energies turned out to be in the range from −7 to 3 kJ/mol (Figure 8), and if analyze the δ^W^(Δ*G*_298_) values (red circles), then only for two conformers (c_23 and c_14), they are positive—i.e., for these conformers, the energy differences increase compared to c_21.

Thus, although the inclusion of the aqueous environment implicitly does not lead to the stabilization of those conformations of 1,3-propanediol that are unstable in vacuum, for most conformers, it decreases their relative energies compared to the most stable conformer.

### 3.2. Determination of Conformation Ratios Based on Radial Distribution Functions

The peak positions in the radial distribution functions *g*_OO_(*r*), obtained in modeling systems with different numbers of molecules, are in excellent agreement with each other (Figure 5), but there are only three peaks, while the conformations of 1,3-propanediol can belong to one of four families with different ranges of intramolecular O…O distances (Figure 4). In addition, the first peak, which corresponds to the GG′ family in terms of distance, has a clearly larger area fraction than follows from the calculations of the conformations by the method proposed in this work (based on the coordinates of all atoms) (Table 5). This discrepancy has a simple explanation.

**Figure 9:**
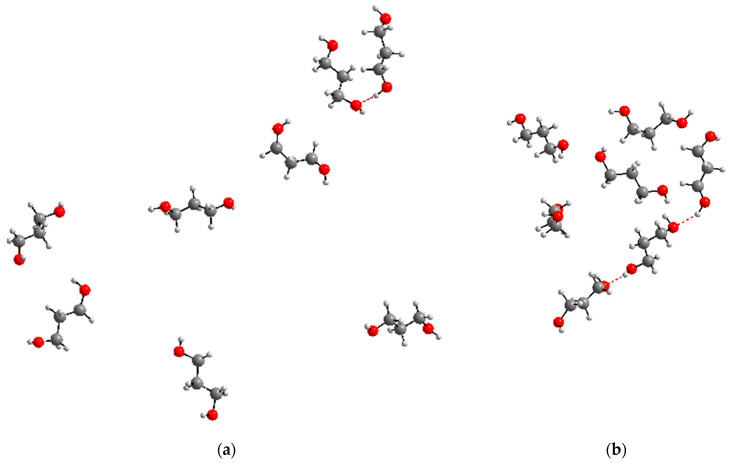
Examples of the formation of intermolecular hydrogen bonds between 1,3-propanediol molecules (indicated by the red dashed line) in the systems under consideration: snapshot for the 800_tTTt system (**a**), part of the snapshot for the 2700_cryst system (**b**) (water molecules not shown for clarity).

Despite the fact that the number of 1,3-propanediol molecules in the systems under consideration is small, they can approach each other with the formation of intermolecular hydrogen bonds (Figure 9).

Thus, the first peak in Figure 4 reflects not so much the fraction of the GG′ conformation family, as the proportion of molecules participating in intermolecular H-bonds. For the GG family, the peak in this figure is absent since the fraction of molecules with such conformations is as small as for GG′ (Table 5), but there is no contribution from favorable intermolecular arrangements. The *g*_OO_(*r*) curves may be described by three Lorentzian or Gaussian functions and after that the area per each peak may be estimated. Depending on the functions (Lorentzians or Gaussians) and the number of molecules in the systems, this gives 7–9% for the first peak, 23–32% for the second, and 59–70% for the third peak. The values for the second and third peaks qualitatively coincide with the calculation results for the TG and TT families (Table 5), though the results for the GG′ family are greatly overestimated. However, it is obvious that with an increase in the concentration of 1,3-propanediol, the tendency to form intermolecular bonds will increase, and accordingly, the accuracy of determining the conformational distribution of molecules based on *g*_OO_(*r*) will deteriorate. Other functions *g*(*r*)—for example, for the distances between oxygen and hydrogen of hydroxyl groups—are less characteristic than *g*_OO_(*r*), and at the same time have the same drawback: they do not allow us to separate the conformations of molecules with intramolecular H-bonds and the formation of intermolecular H-bonds.

The results presented in this section clearly demonstrate that the use of radial distribution functions does not give a reliable evaluation of the conformational distribution of flexible molecules in cases where there is a possibility of forming both intermolecular and intramolecular hydrogen bonds involving the same set of functional groups. In addition, as already noted, the assessment of conformations based on radial distribution functions does not allow the study of the lifetimes of individual conformations.

### 3.3. Lifetimes of Individual Conformations and Families of Conformations

For the most common conformations (all conformations of the TT family), *t*_av_ were in a very narrow range (3.4–3.6 ps) (Table 5). In the rest of the families, the values occurred in a wider range (2–5 ps). The shortest average lifetimes (1–2 ps) were found among conformations of the GG′ family (c_20, c_24, c_25), which were unfavorable or unstable according to quantum chemical calculations, i.e., these conformations are not favorable in dilute aqueous solutions either.

The values of the maximum lifetimes of individual conformations obtained for different systems are in a much wider range, while a tendency to a decrease in *t*_max_ in the sequence of families TT > TG > GG can be noted (Figure 6). In the GG′ family, in all systems, the longest lifetimes were recorded for conformations with intramolecular hydrogen bonds (c_21 [tGG′g] and c_23 [gGG′g]). An increase in the number of considered configurations of 1,3-propanediol molecules in the systems generally contributes to the appearance of longer *t*_max_ for these two conformations. However, in the 800_cryst system (40,000 configurations) for the c_21 conformation, the same *t*_max_ (33 ps) as in the 2700_cryst and 6400_cryst (32 ps) systems with a much larger number of configurations (135,000 and 128,000 respectively) was found.

Under the conditions considered, the average lifetimes of the families were an order of magnitude or greater than the average lifetimes of the individual conformations. The TT family had the longest average lifetime (~110 ps). In the TG family, the average lifetime was about 3 times shorter (31–36 ps), and although most likely coincidental, the fraction of conformations of this family in solution was 3 times less. The shortest lifetimes were in the GG′ (7–16 ps) and GG (11–22 ps) families.

Generally, the mean lifetimes of individual conformations and families were in good agreement with descriptions of the dielectric spectra of propanediols [30,31] and some other diols and their solutions [32] as sums of three or four Debye regions. With such a description, the highest-frequency region corresponds to relaxation times of several (~5) ps. The results of the detailed determination of conformations according to the data from the molecular dynamics simulated in this work support the assumption that this region is due to the rotation of hydroxyl groups and the next (lower frequency) region is due to a change in the main chain of the molecules.

## 4. Materials and Methods

Quantum chemical calculations were performed using the Gaussian 09 program [33]. The values of all torsion angles in the initial approximations of conformations had standard values (180 or ±60°). Imaginary frequencies in conformers obtained from these initial approximations by the MP2/aug-cc-pVDZ and MP2/aug-cc-pVTZ methods were absent in most cases, except for tGG′t (c_20). It was possible to obtain this conformer without imaginary frequencies in two ways: by calculating the force constants at every optimization step or by introducing distortions into the symmetry of the initial approximation (for example, by setting the C2C3O2H2 angle to 175°).

**Figure 10:**
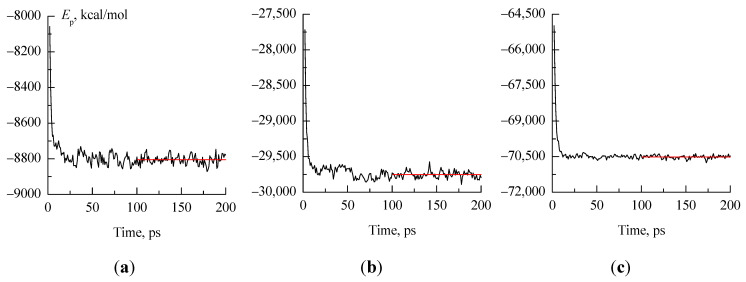
Potential energy values (*E*_p_, kcal/mol) for systems 800_H-b_1 (**a**), 2700_cryst (**b**), 6400_cryst (**c**) in the first 200 ps after the start of molecular dynamics simulations. The red lines demonstrate the stability of the mean values of *E*_p_ after 100 ps.

Four variants of 1,3-propanediol conformations (Figure 1) were used in this work as starting points for molecular dynamics simulations. To create one initial approximation, one molecule of 1,3-propanediol, having one of these conformations, was surrounded by 99 water molecules in the HyperChem 8.0 [34,35] program. A cubic box containing 100 molecules was then enlarged by 2, 3, or 4 times in each dimension (i.e., 8, 27, or 64 times by volume) in the LAMMPS [36] program, which was used to perform molecular dynamics simulations with periodic boundary condition. The *NPT* ensemble was used in all simulations with *P* = 1 atm and *T* = 298.15 K. 1,3-propanediol was described using the OPLS-AA force field [37,38] and TIP4P-Ew model [39] was applied for water. All the considered systems quickly (after no more than 100 ps) reached the equilibrium state (Figure 10). The atomic coordinates of 1,3-propanediol molecules were recorded every 1 ps.

The density of the solution was measured using a VIP-2MR vibrating tube densitometer. Distilled deionized water was used for its calibration and solution preparation. 1,3-propanediol was distilled in a vacuum immediately before the solution was prepared by the gravimetric method (with an accuracy of 0.01 mol %). Measurements were performed for three portions of the solution; the temperature stability was within 0.01 °C.

## 5. Conclusions

Thus, in this work, for the first time, a direct and detailed determination of molecular conformations along molecular dynamics trajectories was carried out that made it possible to establish the relationship between various conformations of 1,3-propanediol in a dilute aqueous solution under normal conditions and to determine the average lifetimes of both individual conformations and their families. It is shown that the proposed method for determining the ratio of conformations and their lifetimes gives results that are perfectly reproduced for different choices of initial approximations and model box sizes.

It was found that the fraction of 1,3-propanediol molecules having an intramolecular hydrogen bond in dilute aqueous solution is extremely insignificant (<0.5%), and this is in good agreement with the conclusions [5] on the tendency of 1,3-butanediol molecules to form intermolecular hydrogen bonds in dilute aqueous solution.

The obtained values of the lifetimes agree well with dielectric spectroscopy data, and they confirm the assumption that the highest-frequency region in the dielectric spectra with a relaxation time of ~5 ps is due to the rotation of hydroxyl groups.

Additionally, using the MP2/aug-cc-pVDZ and MP2/aug-cc-pVTZ methods, it was shown that the 1,3-propanediol molecule in the free state has 22 conformers, and, as was previously established, the most advantageous of them have an intramolecular hydrogen bond. The inclusion of the aqueous environment implicitly does not lead to the stabilization of those conformations of 1,3-propanediol that are unstable in vacuum, but for most conformers, it decreases their relative energies compared to the most stable conformer.

## Figures and Tables

**Table 1:** Relative energies (Δ*E*_el_, Δ*E*_0_ and Δ*G*_298_ in kJ/mol) for 1,3-propanediol conformers optimized from designated conformations in vacuum and in implicit water using PCM model.

Conformation *	In Vacuum						In Water (PCM Model)		
	MP2/aug-cc-pVDZ			MP2/aug-cc-pVTZ			MP2/aug-cc-pVDZ		
	Δ*E*_el_	Δ*E*_0_	Δ*G*_298_	Δ*E*_el_	Δ*E*_0_	Δ*G*_298_	Δ*E*_el_	Δ*E*_0_	Δ*G*_298_
tTTt (c_1)	13.25	11.17	8.77	13.39	11.07	8.68	10.30	8.31	5.60
tTTg (c_2)	13.25	11.24	9.07	13.27	11.12	8.92	10.48	8.74	6.37
gTTg (c_3)	13.70	11.74	9.70	13.60	11.61	9.57	10.64	9.21	7.10
gTTg′ (c_4)	14.81	12.66	10.48	14.72	12.52	10.31	10.82	9.09	6.70
tTGt (c_5)	8.69	6.96	5.23	9.06	7.10	5.24	8.53	6.64	4.45
tTGg (c_6)	9.66	7.84	5.96	9.89	7.91	6.00	8.94	7.00	4.63
tTGg′ (c_7)	13.36	11.12	8.87	13.43	11.18	8.99	10.60	8.90	6.93
gTGt (c_8)	9.43	7.56	5.67	9.66	7.69	5.72	8.92	7.08	4.94
gTGg (c_9)	11.98	9.90	7.90	12.12	9.96	7.89	9.43	7.62	5.52
gTGg′ (c_10)	13.46	11.29	9.24	13.42	11.32	9.31	11.07	9.30	7.26
g′TGt (c_11)	10.39	8.62	6.98	10.65	8.74	7.02	8.41	6.78	4.74
g′TGg (c_12)	11.29	9.45	7.76	11.41	9.52	7.82	8.82	7.12	5.08
g′TGg′ (c_13)	14.26	12.05	10.00	14.16	12.04	10.04	10.79	9.38	7.58
tGGt (c_14)	4.64	3.17	1.76	5.13	3.44	1.81	6.98	5.29	3.38
tGGg (c_15)	7.06	5.52	4.21	7.47	5.74	4.25	7.00	5.56	3.87
tGGg′ (c_16)	4.85	4.80	4.04	5.29	5.06	4.01	7.69	6.39	3.13
gGGg (c_17)	10.07	8.51	7.36	10.44	8.75	7.49	7.17	5.63	3.85
gGGg′ (c_18)	6.28	5.91	4.85	6.71	6.17	4.79	7.94	6.57	3.87
g′GGg′ (c_19)	13.77	11.24	9.28	13.67	11.45	9.66	11.03	9.16	7.38
tGG′t (c_20)	23.54	20.66	16.92	23.83	20.83	17.01	16.76	14.29	10.55
tGG′g (c_21)	0	0	0	0	0	0	0	0	0
tGG′g′ (c_22)	transforms to c_21			transforms to c_21			transforms to c_21		
gGG′g (c_23)	0.82	1.12	1.48	0.99	1.28	1.62	0.99	1.60	1.98
gGG′g′ (c_24)	transforms to c_23			transforms to c_23			transforms to c_23		
g′GG′g (c_25)	transforms to c_21			transforms to c_21			transforms to c_21		

* Conformation numbers used in this work are given in parentheses.

**Table 2:** Some geometric parameters for the 1,3-propanediol molecule in the crystal (QATTEK) and the gGGg′ conformer optimized by MP2/aug-cc-pVDZ and MP2/aug-cc-pVTZ methods.

Parameters	QATTEK	MP2/aug-cc-pVDZ	MP2/aug-cc-pVTZ
H1O1C1C2, °	67.6	65.2	65.3
O1C1C2C3, °	61.5	46.2	47.1
C1C2C3O2, °	69.4	49.1	50.3
C2C3O2H2, °	−82.5	−76.0	−76.4
O1…O2, Å	3.72	3.14	3.16
O1…H2–O2, °	105.6	118.9	117.9

**Table 3:** Main parameters of considered systems: short notation, the total number of molecules in boxes with periodic boundary conditions (*N*), number of 1,3-propanediol molecules (*N*_13PD_), the initial conformation of 1,3-propanediol, simulation time after equilibration (*t*), number of 1,3-propanediol conformations under consideration (*N*_conf_).

System Notation	*N*	*N*_13PD_	Initial Conformation of 13PD	*t*, ns	*N*_conf_
800_cryst	800	8	QATTEK (crystal variant of gGGg′)	5	40,000
800_H-b_1	800	8	tGG′g (opt_MP2/aug-cc-pVDZ)	5	40,000
800_H-b_2	800	8	gGG′g (opt_MP2/aug-cc-pVDZ)	5	40,000
800_tTTt	800	8	tTTt (opt_MP2/aug-cc-pVDZ)	15	120,000
2700_cryst	2700	27	QATTEK (crystal variant of gGGg′)	5	135,000
6400_cryst	6400	64	QATTEK (crystal variant of gGGg′)	2	128,000

**Table 4:** Calculated and experimental density values for 1 mol. % 1,3-propanediol aqueous solution at 298.15 K and atmospheric pressure.

System	Trajectory Length, ns	Step, ps	Density, g/cm^3^
800_cryst	5	1	0.9978 ± 1 × 10^−4^
800_H-b_1	5	1	0.9979 ± 1 × 10^−4^
800_H-b_2	5	1	0.9978 ± 9 × 10^−5^
800_tTTt	15	1/0.1	0.9979 ± 5 × 10^−5^/2 × 10^−5^
2700_cryst	5	1/0.1	0.9977 ± 5 × 10^−5^/2 × 10^−5^
6400_cryst	2	1/0.1	0.9977 ± 5 × 10^−5^/2 × 10^−5^
Experiment (this work)			0.9994 ± 3 × 10^−5^
Experiment [27] (*x*_13PD_ = 1.03 mol. %)			0.9999 ± 3 × 10^−6^

**Table 5:** Contents (φ, % of the numbers of all conformations during simulations) of 1,3-propanediol conformations with different designations and their average lifetimes (*t*_av_, ps *) in systems under consideration.

Conformation	Family	800_cryst		800_H-b_1		800_H-b_2		800_tTTt		2700_cryst		6400_cryst	
		φ	*t*_av_	φ	*t*_av_	φ	*t*_av_	φ	*t*_av_	φ	*t*_av_	φ	*t*_av_
tTTt (c_1)	TT	15.5	3.6	16.1	3.6	15.1	3.6	15.2 **	*3.5*	15.6	3.5	15.5	3.6
tTTg (c_2)		36.8	3.5	38.7	3.5	37.2	3.6	37.9	3.6	38.0	3.5	37.8	3.5
gTTg (c_3)		11.1	3.6	11.9	3.5	11.1	3.6	11.1	3.4	11.3	3.4	11.6	3.6
gTTg′ (c_4)		10.5	3.5	11.1	3.4	10.5	3.6	10.5	3.4	10.7	3.4	11.0	3.5
tTGt (c_5)	TG	5.2	3.2	4.7	3.5	5.3	3.3	5.0	3.4	5.1	3.6	5.4	3.6
tTGg (c_6)		3.6	4.3	4.0	4.3	4.0	3.9	4.0	4.0	3.8	4.2	3.6	3.9
tTGg′ (c_7)		2.2	2.7	1.7	2.8	1.9	2.8	1.8	2.8	1.8	2.8	2.0	2.7
gTGt (c_8)		3.7	3.5	2.7	3.5	3.0	3.3	3.3	3.5	3.2	3.5	3.2	3.4
gTGg (c_9)		2.0	4.0	1.8	3.8	2.4	4.7	2.2	3.8	2.4	4.3	2.2	4.0
gTGg′ (c_10)		1.3	2.7	0.9	2.5	1.1	2.8	1.1	2.5	1.0	2.6	1.2	2.6
g′TGt (c_11)		3.5	3.5	2.6	3.3	3.3	3.5	3.2	3.3	2.9	3.3	2.7	3.1
g′TGg (c_12)		2.4	4.0	2.3	4.0	2.7	3.9	2.6	4.0	2.1	3.6	2.1	3.7
g′TGg′ (c_13)		1.0	2.3	0.8	2.3	1.0	2.5	1.2	2.7	1.0	2.8	1.0	2.6
tGGt (c_14)	GG	0.1	2.9	0.05	1.7	0.1	2.9	0.1	3.4	0.1	3.1	0.1	2.8
tGGg (c_15)		0.1	2.6	0.2	3.0	0.4	4.6	0.1	3.5	0.1	3.9	0.1	3.1
tGGg′ (c_16)		0.1	2.4	0.1	3.2	0.1	2.6	0.1	3.0	0.1	3.0	0.1	2.8
gGGg (c_17)		0.04	4.5	0.04	7.0	0.1	2.5	0.1	4.7	0.02	2.9	0.03	2.7
gGGg′ (c_18)		*0.05*	*2.5*	0.1	2.6	0.1	3.3	0.1	2.3	*0.1*	*2.3*	*0.05*	*2.9*
g′GGg′ (c_19)		0.02	1.8	0.002	1	0.05	5.0	0.01	1.9	0.02	2.1	0.005	2.3
tGG′t (c_20)	GG′	0.01	2.0	0	0	0.005	2	0.01	3.0	0.01	1.4	0.004	1.0
tGG′g (c_21)		0.3	4.0	*0.05*	*4.0*	0.1	4.3	0.2	4.8	0.2	6.2	0.1	5.8
tGG′g′ (c_22)		0.1	2.3	0.01	1.5	0.03	6.5	0.03	2.0	0.02	1.4	0.01	1.5
gGG′g (c_23)		0.1	3.3	0.04	2.3	*0.1*	*3.1*	0.1	4.6	0.1	4.1	0.1	3.7
gGG′g′ (c_24)		0.02	1.5	0	0	0.01	1.7	0.01	1.8	0.01	2.0	0.01	1.5
g′GG′g (c_25)		0.03	1.5	0.01	1.5	0.005	1.0	0.01	1.4	0.01	1.4	0.01	1.1

* For conformations that appeared only once in particular systems, the time is given without decimals. ** Data for conformations that were the starting points in particular systems are in italics.

**Table 6:** Maximum lifetimes (*t*_max_, ps) of 1,3-propanediol conformations with different designations in systems under consideration (data for conformations that were the starting points in particular systems are in italics).

Conformation	Family	800_cryst	800_H-b_1	800_H-b_2	800_tTTt	2700_cryst	6400_cryst
tTTt (c_1)	TT	31	29	35	*34*	36	34
tTTg (c_2)		48	38	41	45	43	48
gTTg (c_3)		42	33	33	32	49	39
gTTg′ (c_4)		42	31	38	43	59	47
tTGt (c_5)	TG	28	31	26	28	36	32
tTGg (c_6)		34	27	26	33	43	33
tTGg′ (c_7)		22	23	17	23	22	25
gTGt (c_8)		32	33	20	28	21	37
gTGg (c_9)		25	26	27	40	39	49
gTGg′ (c_10)		29	18	20	21	16	21
g′TGt (c_11)		40	27	22	27	23	30
g′TGg (c_12)		25	23	26	46	34	30
g′TGg′ (c_13)		14	15	17	29	17	19
tGGt (c_14)	GG	9	4	18	14	14	11
tGGg (c_15)		7	14	22	17	13	16
tGGg′ (c_16)		8	14	8	13	16	13
gGGg (c_17)		6	8	7	32	11	10
gGGg′ (c_18)		*9*	6	7	7	*6*	*7*
g′GGg′ (c_19)		3	1	13	5	10	4
tGG′t (c_20)	GG′	3	0	2	4	3	1
tGG′g (c_21)		33	*9*	15	18	32	32
tGG′g′ (c_22)		7	2	12	6	6	5
gGG′g (c_23)		7	8	*15*	22	14	19
gGG′g′ (c_24)		3	0	3	4	6	6
g′GG′g (c_25)		4	2	1	3	3	2

**Table 7:** Average lifetimes (ps) for the conformation families of 1,3-propanediol.

Family	800_cryst	800_H-b_1	800_H-b_2	800_tTTt	2700_cryst	6400_cryst
TT	103	124	116	110	108	110
TG	32	32	36	33	31	32
GG	13	11	22	16	13	12
GG′	12	7	16	14	12	11

## Data Availability

The original contributions presented in this study are included in the article. Further inquiries can be directed to the corresponding author.
